# Associations between organizational cynicism, workplace alienation, and nurses’ work effectiveness: A cross-sectional questionnaire survey

**DOI:** 10.1016/j.ijnsa.2026.100588

**Published:** 2026-06-03

**Authors:** Ali D. Abousoliman, Hagar Mahmoud Hamed

**Affiliations:** a College of Nursing, Prince Sattam bin Abdulaziz University, Al-Kharj, Saudi Arabia; b Faculty of Nursing, KafrElsheikh University, Egypt

**Keywords:** Organizational cynicism, Workplace alienation, Work effectiveness, Nursing management, Hospital administration

## Abstract

**Background:**

The effectiveness of the nursing workforce is critical for patient safety and quality of care, yet nurses often face high-stress environments that can foster negative psychosocial states. Although organizational cynicism and workplace alienation are recognized as important workplace concerns, their concurrent associations with nurses' work effectiveness remain underexplored, particularly in non-Western health care contexts.

**Aim:**

This study aimed to assess both organizational cynicism and workplace alienation among staff nurses and explore their separate and concurrent associations with perceived work effectiveness.

**Methods:**

This descriptive correlational study used convenience sampling to recruit 218 RNs from a university-based hospital in Egypt. Data were gathered using three standardized instruments: the Organizational Cynicism Scale, the Workplace Alienation Scale, and the Conditions for Work Effectiveness Questionnaire. Data was analyzed using frequency, *t*-test, ANOVA, Pearson correlation, and linear multiple regression.

**Results:**

Nurses reported moderate levels of organizational cynicism and workplace alienation, alongside relatively high levels of perceived work effectiveness. Both organizational cynicism and workplace alienation were significantly and negatively correlated with work effectiveness. A strong positive correlation was observed between cynicism and alienation. In the multiple regression model, workplace alienation emerged as a significant negative predictor of work effectiveness, whereas organizational cynicism did not retain statistical significance when both variables were considered simultaneously. Collinearity diagnostics indicated acceptable levels of multicollinearity.

**Conclusion:**

The findings suggest that while organizational cynicism and workplace alienation are closely related and both associated with diminished work effectiveness, alienation represents a more proximal factor linked to nurses’ perceived effectiveness. Addressing workplace alienation through strategies that enhance autonomy, meaning, and social integration may be critical for strengthening nursing work effectiveness.

**What is already known**

Organizational cynicism and workplace alienation are recognized as negative psychological states associated with employee attitudes like job satisfaction and commitment.

Nurse well-being and the quality of the practice environment are established correlates to patient outcomes and overall organizational performance.

Alt-text: Unlabelled box dummy alt text

**What this paper adds**

This study provides empirical evidence on the associations between organizational cynicism, workplace alienation, and nurses’ self-perceived work effectiveness.

Findings suggest that these negative psychosocial states are relevant organizational concerns that may be associated with lower perceived nursing work effectiveness.

The results offer nurse leaders evidence to consider strategies aimed at reducing workplace alienation and organizational cynicism and promoting a more supportive work environment.

Alt-text: Unlabelled box dummy alt text

## 1 Introduction

In contemporary health care systems, human capital remains a decisive determinant of care quality, patient safety, and organizational sustainability. Nursing effectiveness is particularly critical because nurses are expected to provide continuous, complex, and patient-centered care that directly influences clinical outcomes (Aiken et al., 2024). Nurses are expected to mobilize professional judgment, information, resources, and interprofessional collaboration to deliver high-quality care. However, increasing workload pressures, administrative complexity, staffing shortages, and structural constraints expose nurses to substantial psychosocial strain that may compromise their work functioning (Yu et al., 2025).

Among the psychosocial factors associated with nursing practice, organizational cynicism and workplace alienation have attracted growing scholarly attention. Organizational cynicism is characterized by distrust, negative affect, and critical behaviors directed toward the employing organization. Workplace alienation is a related form of psychological estrangement from work, reflected in feelings of powerlessness, meaninglessness, normlessness, and social isolation (Vohra et al., 2019). While the two constructs are conceptually connected, cynicism represents an evaluative attitude toward the organization, whereas alienation reflects a more pervasive disconnection from the work role.

These constructs can be understood through established theoretical perspectives. Social Exchange Theory posits that workplace attitudes develop based on perceived reciprocity and fairness within the employment relationship (Ahmad et al., 2023). Perceived violations of the psychological contract may foster distrust and critical attitudes toward the organization, contributing to cynicism. Similarly, the Job Demands–Resources (JD-R) model explains how excessive job demands combined with insufficient resources (e.g., autonomy, support, role clarity) may lead to disengagement and deteriorating psychological attachment to work (Schaufeli, 2017). Within this framework, alienation may represent an outcome of sustained resource deprivation and diminished professional control.

Empirical evidence has associated both cynicism and alienation to adverse outcomes such as burnout, reduced engagement, turnover intentions, and lower perceived performance. However, most studies examine these constructs independently and focus on discrete outcomes rather than broader conceptualizations of work effectiveness. Work effectiveness particularly when conceptualized from a structural empowerment perspective extends beyond task completion and encompasses access to information, resources, support, and opportunities necessary for high-quality professional performance (Vafeas et al., 2025). The extent to which organizational cynicism and workplace alienation uniquely and jointly associated with this broader construct remains insufficiently explored, especially in non-Western health care contexts. Therefore, the aim of this study was to examine the levels of organizational cynicism and workplace alienation among staff nurses and to examine their individual and combined associations with nurses’ work effectiveness. By examining the relative and overlapping associations of these constructs, this study seeks to advance understanding of negative workplace attitudes in nursing and inform evidence-based managerial strategies aimed at strengthening workforce effectiveness.

### 1.1 Review of literature and hypotheses

Organizational cynicism and workplace alienation can be understood within broader frameworks explaining employee organization relationships. Social Exchange Theory suggests that employee attitudes are shaped by perceived reciprocity, fairness, and mutual obligation (Ahmad et al., 2023). When nurses perceive imbalance between their contributions and organizational returns, distrust and critical attitudes toward the organization may develop, fostering organizational cynicism (Bell, 2011). The Job Demands–Resources model further explains how workplace stressors are associated with psychological functioning (Schaufeli, 2017). According to this model, excessive job demands combined with insufficient resources such as autonomy, supervisory support, opportunities for development, may result in disengagement and deteriorating work attitudes. While cynicism may emerge as a defensive evaluative stance toward the organization, workplace alienation may reflect a more profound psychological state characterized by estrangement from one’s work role and diminished sense of meaning or control (Healy and Wilkowska, 2017).

Empirical studies indicate that organizational cynicism among nurses is associated with burnout, emotional exhaustion, reduced teamwork, and lower perceived performance (Ike et al., 2024). Cynical attitudes may weaken organizational commitment and reduce constructive engagement in workplace processes essential for effective patient care delivery. From a structural empowerment perspective, effective nursing practice depends on access to information, resources, support, and opportunities for professional growth (Vafeas et al., 2025). Cynicism may undermine nurses’ willingness to fully engage with these structures, thereby being associated with lower perceived work effectiveness. This suggests the following hypothesis was formulated: *H1: Organizational cynicism is negatively related to perceived work effectiveness.*

Workplace alienation is particularly relevant in hierarchical and bureaucratic hospital systems, where nurses may experience limited autonomy or restricted participation in decision-making processes (Şekerli, 2025; Abd-Elrhaman et al., 2020). Feelings of powerlessness and meaninglessness may reduce intrinsic motivation, initiative, and proactive professional engagement. Unlike cynicism, which primarily reflects evaluative distrust toward the organization, alienation represents a more pervasive psychological disconnection from work itself.

Research links alienation to decreased motivation, lower engagement, and stronger turnover intentions (Belanger et al., 2024). Because effective nursing performance requires psychological engagement and a sense of professional agency, alienation may directly impair nurses’ perceived ability to function effectively. So, the following hypothesis was formulated: *H2: Work alienation is negatively related to perceived work effectiveness.*

Although both constructs are negatively related to work outcomes, prior research rarely examines their concurrent associations within the same analytical model. Given their conceptual relatedness, some degree of overlap in their associations is expected. However, theoretical distinctions suggest they may not show equivalent associations with work effectiveness. Cynicism reflects a critical evaluation of the organization, whereas alienation reflects experiential estrangement from work itself. Because work effectiveness is inherently tied to engagement with the work role, alienation may show a comparatively stronger association. Nonetheless, limited empirical evidence has examined how organizational cynicism and workplace alienation are concurrently associated with nurses’ work effectiveness, particularly in non-Western health care contexts. The following hypotheses were formulated: *H3: Organizational cynicism and workplace alienation are jointly associated with nurses’ perceived work effectiveness. H4: Workplace alienation is negatively associated with nurses’ work effectiveness after accounting for organizational cynicism.*

### 1.2 Significance of the study

Despite the evidence that both cynicism and alienation are associated with lower effectiveness of a healthy nursing workforce, research is scattered, with very limited studies investigating either of these concepts individually (Durrah, et al., 2023; Yıldız, and Şaylıkay, 2014). Similarly, there is little research that explores their individual and collective associations with work effectiveness in a broad-based construct, especially in developing or non-Western nations, where the potential moderating role of organizational or cultural factors could shape these attitudes. Thus, there is a need for research that focuses on both cynicism and alienation with respect to work effectiveness among nurses. This study addresses this gap by examining organizational cynicism, workplace alienation, and nurses’ work effectiveness within an Egyptian hospital context.

### 1.3 Study objectives

• Assess the current levels of organizational cynicism, workplace alienation, and work effectiveness among nursing staff in Kafr El-Sheikh University Hospitals, Egypt.
• Examine the associations among organizational cynicism, workplace alienation, and nurses' work effectiveness.
• Determine the extent to which organizational cynicism and workplace alienation, individually and collectively, are associated with variations in nurses’ work effectiveness.

## 2 Methods

### 2.1 Study design

A descriptive, cross-sectional correlational research design was employed.

### 2.2 Setting and sample

This study was carried out in November 2025, at Kafr El-Sheikh University Hospitals, a 474-bed university hospital in Kafr El-Sheikh City (population ≈ 3.8 millions), Egypt. A convenience sampling method was used, targeting registered nurses working across multiple clinical departments (e.g., medical, surgical, and critical care units). No inclusion or exclusion criteria were applied based on department, if nurses met the general eligibility requirements. Selection criteria involved being a full-time employee with no less than one year of experience at the organization. Nurses with less than one year of experience were excluded.

Data were collected using a self-administered questionnaire distributed in person during work shifts after obtaining administrative approval. Participation was voluntary, and questionnaires were completed anonymously.

### 2.3 Data collection instruments

A self-report questionnaire with three standardized scales was employed. Due to the Egyptian context, the translation of English-language scales to Arabic involved Brislin translation techniques with back-translation to establish semantic/conceptual equivalence.

### 2.4 Tool I: organizational cynicism scale (OC)

Brandes et al. (1999) designed the 14-item scale to measure cynicism in organizations, using three scales: cognitive cynicism (5 items), which refers to employees’ beliefs that the organization lacks honesty, fairness, or integrity; affective cynicism (4 items), which reflects negative emotions toward the organization, such as anger, frustration, or disappointment; and behavioral cynicism (5 items), which refers to critical or disparaging behaviors directed toward the organization. Personal demographics such as age, gender, work experience, and educational levels were gathered. A 5-point Likert scale response system was used (1 = Strongly Agree to 5 = Strongly Disagree), with higher scores representing more cynicism. indicating greater levels of cynicism. For interpretive purposes, raw scores were converted into percentage scores by dividing the obtained total score by the maximum possible score and multiplying by 100. Percentage ranges were then categorized as follows: >75% = high, 60–75% = moderate, and <60% = low levels of cynicism.

### 2.5 Tool II: workplace alienation scale (WA)

This scale of 39 items was developed by Mohammed & Hassanein (2017) for testing five constructs of alienation: self-estrangement (9 items), powerlessness (7 items), meaninglessness (7 items), normlessness (8 items), and social isolation (8 items). A 5-point Likert scale was used for responding to the statements ranging from 1 (Strongly Disagree) to 5 (Strongly Agree). from 1 (Strongly Disagree) to 5 (Strongly Agree). For interpretive purposes, total and subscale scores were converted into percentage scores by dividing the obtained score by the maximum possible score for each scale and multiplying by 100. Based on these percentage scores, perception levels were classified as follows: >75% = highly perceived, 60–75% = moderately perceived, and <60% = low.

### 2.6 Tool III: conditions for work effectiveness questionnaire

This is a 29-item scale, developed by Elsawy (2023) based on Laschinger et al. (2004), to assess the work effectiveness of nurses. This scale contains four factors: Access to Opportunity (7 items), Access to Information (9 items), Access to Support (5 items), and Access to Resources (8 items). This instrument employed a 3-point frequency scale (1 = Never, 2 = Sometimes, 3 = Always). Percent scores were calculated, indicating levels of effectiveness: scores >75% indicated a high level of work effectiveness, scores from 60% to 75% indicated a moderate level, and scores <60% indicated a low level of work effectiveness.

### 2.7 Procedure and ethical considerations

This research was approved by The Scientific Research Ethics Committee of Kafr El-Sheikh University with approval No (KFSIRB200–433). Additionally, Kafr El-Sheikh University Hospital administration gave permission to carry out the research among their employees. Participants were informed about the study’s objectives. A consent form was attached to the research instruments, namely, the questionnaires that were distributed to the research targets in sealed envelopes. Moreover, responses were collected directly from the participants to ensure privacy and confidentiality. Questionnaires were completed individually by nurses without the presence of supervisors or managers and were returned in sealed envelopes to the researcher. Submission of the completed questionnaire was considered implicit informed consent to participate in the study.

### 2.8 Statistical analysis

Data analysis was carried out using SPSS V27. Descriptive statistics such as means, standard deviations, and frequencies were employed in data description for demographics and scales. *t*-tests and one-way ANOVA were utilized for comparing the differences among various demographics. Pearson’s correlation was used to establish association between various key variables. Multiple linear regression analysis was used to examine whether organizational cynicism and workplace alienation were associated with work effectiveness when considered together. Work effectiveness was entered as the dependent variable, while organizational cynicism and workplace alienation were entered as independent variables. Significance levels were set at p < .05.

### 2.9 Pilot study

To ensure the methodological soundness of this research, a preliminary pilot study was conducted to validate the data collection instrument and refine the research protocol. We recruited a convenience sample of 30 registered nurses from an external hospital facility to complete the draft questionnaire. This exercise was critical for evaluating the instrument's face validity, confirming the internal consistency of the scales, and establishing a reliable estimate for completion time. The analysis yielded strong psychometric properties, with Cronbach's Alpha coefficients for the Organizational Cynicism (α = 0.88), Workplace Alienation (α = 0.91), and Work Effectiveness (α = 0.86) scales all demonstrating excellent reliability. In response to participant feedback, we implemented minor but crucial linguistic refinements to two items to enhance clarity. Furthermore, the pilot confirmed an average completion time of 12 min, a vital metric for our main data collection strategy. This preparatory phase was instrumental in optimizing the instrument and ensuring the integrity and feasibility of the subsequent large-scale investigation.

## 3 Results

A total of 218 staff nurses’ responses were included in the analysis. As shown in Table 1, the sample included nurses from multiple clinical departments, with the majority being female and representing a range of age groups, marital statuses, and years of work experience. Differences across demographic variables indicated that age was significantly associated with both organizational cynicism and workplace alienation. Younger nurses (aged <30 years) reported higher mean scores for organizational cynicism (M = 38.72; F = 5.081, p = .007) and workplace alienation (M = 107.36; F = 7.740, p = .001) compared with older nurses. Marital status was also significant, with single nurses reporting higher levels of organizational cynicism (F = 3.066, p = .049) and workplace alienation (F = 3.233, p = .041) compared to married nurses.

**Table 1:** Differences in Organizational Cynicism, Workplace Alienation, and Nurses’ Work Effectiveness by Participants’ Demographic Characteristics (N = 218).

		**N**	**Organizational Cynicism**			**Workplace Alienation**			**Work Effectiveness**		
			**Mean**	**S. D**	**Test of sig.**	**Mean**	**S. D**	**Test of sig.**	**Mean**	**S. D**	**Test of sig.**
**Age**	<30	133(61%)	38.72	11.04	F = 5.081 P=.007	107.36	34.77	F = 7.740 P=.001	65.69	15.09	F = 1.492 P=.227
	from 31–40	77(35.3%)	34.83	9.76		95.20	26.43		67.53	10.32	
	≥40	8(3.7%)	29.75	14.90		69.87	24.13		73.37	11.80	
**Gender**	Male	54(24.8%)	38.03	13.26	T = 0.687 P=.494	103.57	43.10	T = 0.398 P=.692	64.61	17.35	T=−1.262 P=.208
	Female	164(75.2%)	36.68	10.11		101.07	28.63		67.28	11.99	
**Marital Status**	Single	49(22.5%)	40.24	12.47	F = 3.066 P=.049	111.97	39.50	F = 3.233 P=.041	63.46	16.41	F = 2.516 P = 0.083
	Married	153(70.2%)	35.88	10.08		98.46	28.42		67.95	12.01	
	Divorced	16(7.3%)	38.00	12.68		101.06	43.10		63.56	16.23	
**Educational Level**	Diploma	120(55%)	36.66	10.39	F = 0.137 P=.872	99.83	31.78	F = 0.483 P=.618	67.95	13.08	F = 1.293 P=.277
	Bachelor	80(36.7%)	37.46	11.48		104.48	33.54		65.07	14.56	
	Postgraduate	18(8.3%)	37.38	12.76		101.66	36.22		64.66	11.19	
**Experience Years**	1–5years	114(52.2%)	38.79	11.12	F = 5.183 P=.006	107.71	34.58	F = 10.753 P=.000	65.81	15.47	F = 2.296 P=.103
	6–10 years	69(31.8%)	36.53	9.74		102.89	28.42		65.69	10.33	
	≥11	35(16%)	32.17	11.40		79.71	24.95		71.08	11.58	

No statistically significant differences in work effectiveness were observed by gender (t = −1.262, p = .208), although female nurses reported slightly higher mean levels of work effectiveness (M = 67.28) than male nurses (M = 64.10). Organizational cynicism varied significantly by years of work experience (F = 5.183, p = .006) and workplace alienation also varied significantly by years of work experience (F = 10.753, p < .001), with nurses reporting 1–5 years of experience exhibiting higher levels of both constructs. Educational attainment did not demonstrate statistically significant differences across any of the study variables (Table 1).

Descriptive statistics indicated that nurses experienced moderate levels of both organizational cynicism (M = 37.02, representing 52.9% of the maximum possible score) and workplace alienation (M = 101.69, representing 52.1% of the maximum possible score). Subscale analysis showed that, within organizational cynicism, the cognitive dimension (distrust) was most prominent (M = 14.49, 58%; SD = 4.39). Within workplace alienation, normlessness, which reflects perceptions of unclear or weakened workplace norms and expectations, was the most frequently reported dimension (M = 22.02, 55.1%; SD = 7.86), followed by social isolation (M = 21.53, 53.8%; SD = 7.36). In contrast, overall work effectiveness was relatively high (M = 66.62, 76.6% of the maximum score), indicating that nurses generally perceived adequate access to work-related resources, support, and performance enablers despite the presence of negative workplace attitudes (Table 2).

**Table 2:** Descriptive Statistics for Study Variables.

Variable	Subdimension	Minimum	Maximum	Mean	Std. Deviation
**Organizational** **Cynicism**	Cognitive	5.00	25.00	14.49(58%)	4.39
	Affective	5.00	25.00	13.04(52.2%)	4.86
	Behavioral	4.00	20.00	9.48(47.4%)	3.82
	**Total Cynicism**	**14.00**	**61.00**	**37.01(52.9%)**	**10.96**
**Work Alienation**	Powerless	7.00	31.00	17.20(44.2%)	5.25
	Meaningless	7.00	35.00	17.82(50.9%)	7.07
	Self-estrangement	9.00	44.00	23.10(51.4%)	8.39
	Social isolation	8.00	38.00	21.53(53.8%)	7.36
	Normlessness	8.00	39.00	22.02(55.1%)	7.86
	**Total Alienation**	**39.00**	**182.00**	**101.69(52.1%)**	**32.72**
**Work Effectiveness**	Access to opportunity	7.00	21.00	16.54(78.8%)	3.32
	Access to Support	9.00	27.00	20.53(76.1%)	5.01
	Access to resources	5.00	15.00	11.29(75.3%)	2.73
	Access to information	8.00	24.00	18.25(76%)	4.30
	**Total Effectiveness**	**29.00**	**87.00**	**66.62(76.6%)**	**13.52**

Pearson correlation analysis showed statistically significant associations among the study variables. Both organizational cynicism and workplace alienation were significantly and negatively correlated with work effectiveness (r = −.543 and r = −.618, respectively; p < .01). A strong positive correlation was observed between organizational cynicism and workplace alienation (r = 0.807, *p* < .01), indicating a high degree of association between the two constructs (Table 3).

**Table 3:** Correlation matrix of the study variables.

		**Organizational Cynicism**	**Workplace Alienation**
**Workplace Alienation**	Pearson Correlation	.807^⁎⁎^	1
	Sig. (2-tailed)	.000	
**Work Effectiveness**	Pearson Correlation	−0.543^⁎⁎^	−0.618^⁎⁎^
	Sig. (2-tailed)	.000	.000

As shown in Table 4, multiple linear regression analysis revealed that the overall regression model was statistically significant (F = 67.99, *p* < .001). The model explained 38.7% of the variance in nurses’ work effectiveness (R² = 0.387). In the adjusted model, workplace alienation was significantly and negatively associated with nurses’ work effectiveness (B = −0.213, β = −0.516, t = −5.717, *p* < .001), indicating that higher workplace alienation was associated with lower work effectiveness. Organizational cynicism was not significantly associated with nurses’ work effectiveness after accounting for workplace alienation (B = −0.155, β = −0.126, t = −1.393, p = .165).

**Table 4:** Multiple linear regression analysis of organizational cynicism, workplace alienation, and nurses’ work effectiveness.

**Model**	**Unstandardized Coefficients (B)**	**Standardized Coefficients (Beta)**	***t***	**Sig. (p-value)**
**(Constant)**	94.085		36.390	.000
**Organizational Cynicism**	−0.155	−0.126	−1.393	.165
**Workplace Alienation**	−0.213	−0.516	−5.717	.000

**R=0.622, R² = 0.387** (The independent variables explain 38.7% of the variance in Work Effectiveness). F = 67.99, p < .001. The regression model was statistically significant (p < .001).

Collinearity diagnostics indicated acceptable levels of multicollinearity. Variance inflation factor (VIF) values for both independent variables were 2.864, with corresponding tolerance values of 0.349, suggesting that multicollinearity was not of sufficient magnitude to invalidate the regression estimates.

## 4 Discussion

This study aimed to examine the associations between organizational cynicism, workplace alienation, and nurses’ work effectiveness. The findings indicate that while both constructs are negatively associated with work effectiveness, they showed different patterns of association when examined simultaneously. Specifically, although organizational cynicism demonstrated a significant bivariate association with work effectiveness, its association did not remain significant in the multivariate model once workplace alienation was included. In contrast, workplace alienation showed the stronger and statistically significant negative association with work effectiveness.

The observed negative association between organizational cynicism and work effectiveness aligns with prior research and can be interpreted through the lens of Social Exchange Theory. When nurses perceive their organization as untrustworthy or as failing to fulfill psychological contract obligations, they may reciprocate by withdrawing effort, engagement, or discretionary behaviors that support effective performance (Çivilidağ et al., 2025; Chiaburu et al., 2013). This cognitive–affective stance of distrust may erode intrinsic motivation and reduce willingness to go beyond formal role requirements. Consistent with this interpretation, previous studies have linked organizational cynicism among nurses to burnout and diminished organizational citizenship behaviors (Ejupi and Squires, 2024; Mohamed et al., 2024). However, the regression findings of the present study suggest that the association between cynicism on work effectiveness may be indirect or overlapping, particularly when more pervasive psychological states such as alienation are considered.

A central finding of this study is the robust negative association between workplace alienation and work effectiveness. This result is consistent with the Job Demands–Resources model, which posits that sustained depletion of job resources contributes to disengagement and reduced performance (Demerouti, 2025; Lee and Jo, 2023). Alienation, characterized by feelings of powerlessness, meaninglessness, and social isolation, reflects a profound erosion of psychological resources necessary for effective functioning. Nurses who perceive limited control over their work, experience diminished meaning in their professional roles, or feel socially disconnected may struggle to utilize available structural facilitators of effectiveness, such as information, support, and opportunities for professional contribution (Alfuqaha et al., 2023; Batho, 2022). This result indicates that reduced connection to the meaning and social context of work may be particularly relevant to nurses’ perceived work effectiveness.

The comparative examination of cynicism and alienation offers important theoretical insights. Although these constructs are frequently discussed together, the findings suggest that they may operate through distinct pathways in relation to work effectiveness. Organizational cynicism may represent an earlier or less severe response to perceived organizational shortcomings, whereas alienation may reflect a more entrenched psychological condition that more directly undermines work functioning (Vohra et al., 2019; Yıldız and Şaylıkay, 2014). This interpretation aligns with the perspective advanced by Şekerli (2025) and Ghaleb (2024), who conceptualize alienation as a deeper and more complex psychological state than either job dissatisfaction or cynicism, insofar as it affects the individual’s relationship with their current work role.

These findings contribute to theory by extending the JD–R framework to highlight workplace alienation as a critical outcome of resource depletion with direct implications for performance-related outcomes. Additionally, they refine applications of Social Exchange Theory by suggesting that perceived psychological contract violations may initially manifest as cynicism and, if unresolved, evolve into alienation, signaling a more severe breakdown in the exchange relationship (Kwon and Kim, 2020). This distinction challenges organizational behavior models that treat negative workplace attitudes as a homogeneous construct and underscores the importance of differentiating between evaluative attitudes and deeper states of psychological disengagement.

From a practical perspective, the findings underscore the need for nurse administrators to move beyond surface-level interventions aimed solely at improving morale or trust. Addressing workplace alienation requires targeted strategies that restore nurses’ sense of agency, meaning, and social connection, such as involving nurses in unit-level decision-making, improving role clarity, recognizing nurses’ contributions to patient outcomes, strengthening shared governance, and creating structured opportunities for peer support and mentoring. To mitigate powerlessness, hospital administrators should implement shared governance structures that grant nurses meaningful autonomy in clinical practice and scheduling (Uchechukwu, 2024; Kähkönen et al., 2021). To counteract meaninglessness, leadership efforts should emphasize the broader purpose and impact of nursing work more visible by linking routine nursing activities to patient safety and quality outcomes, sharing patient and family feedback, involving nurses in quality-improvement initiatives, and recognizing specific examples of how nurses’ contributions improve care continuity, comfort, recovery, and patient education. Such efforts may not only reduce feelings of alienation by restoring meaning and professional identity but may also help mitigate organizational cynicism by strengthening nurses’ sense of alignment with organizational values and goals (Belanger et al., 2024). Reducing social isolation may require investment in mentorship programs and team-based work structures, particularly for early-career nurses (Frangieh et al., 2024). Such interventions may alleviate alienation and, in turn, support higher levels of work effectiveness.

The findings should be interpreted within the context of hospital environments characterized by high demands, bureaucratic complexity, and emotionally intensive work. These conditions may simultaneously contribute to the development of cynicism and alienation (Assis et al., 2022). Moreover, while the study was conducted within a specific socio-cultural context, the psychological processes identified may be broadly applicable across health care systems. The demographic findings, indicating heightened vulnerability among younger and less experienced nurses, further reinforce the importance of early-career support mechanisms to mitigate reality shock and promote professional integration (Nakua and Marsh, 2025; Yao and McWha-Hermann, 2025).

## 5 Conclusion

This study provides evidence that workplace alienation is significantly associated with lower nurses’ work effectiveness. While organizational cynicism and demographic characteristics are related to nurses’ workplace perceptions, alienation showed a stronger negative association with perceived effectiveness in the regression model. These findings highlight the importance of addressing structural and relational conditions associated with alienation. Promoting empowerment, shared purpose, and social integration among nursing staff may be important for supporting workforce effectiveness and sustaining high-quality patient care. Because of the cross-sectional correlational design, these findings should be interpreted as associations rather than causal effects.

### 5.1 Limitations and future research

This study has several limitations. The cross-sectional design limits causal inference, and reliance on self-report measures may introduce common method bias. Data were collected from a single hospital in Egypt, which may constrain generalizability to other settings. Future research should employ longitudinal designs to examine how cynicism and alienation evolve over time and are associated with performance trajectories. Qualitative approaches may further illuminate nurses lived experiences of alienation, and the inclusion of objective performance indicators (e.g., patient outcomes or safety metrics) could provide a more comprehensive assessment of work effectiveness.

## Data availability

The data that support the findings of this study are available from the corresponding author upon reasonable request.

## CRediT authorship contribution statement

**Ali D. Abousoliman:** Writing – review & editing, Writing – original draft, Methodology, Investigation, Data curation, Conceptualization. **Hagar Mahmoud Hamed:** Supervision, Resources, Methodology.

## Declaration of competing interest

The authors declare that they have no known competing financial interests or personal relationships that could have appeared to influence the work reported in this paper.

## References

[bib0001] Abd-Elrhaman E.A., Helal W.H., Araby-Ebraheem S.M. (2020). Organizational justice, work alienation and deviant behaviors among staff nurses. Int. J. Nurs. Didact..

[bib0002] Ahmad R., Nawaz M.R., Ishaq M.I., Khan M.I., Ashraf H.A. (2023). Social exchange theory: systematic review and future directions. Front. Psychol..

[bib0003] Aiken L.H., Sermeus W., McKee M. (2024). Physician and nurse well-being, patient safety and recommendations for interventions: cross-sectional survey in hospitals in six European countries. BMJ Open..

[bib0004] Alfuqaha O.A., Shunnar O.F., Khalil R.A., Alhalaiqa F.N., Thaher Y.A., Al Amad T.Z. (2023). Work alienation influences nurses’ readiness for professional development and willingness to learn: a cross-sectional correlation study. PLoS. One.

[bib0006] Assis B.B., Azevedo C., Moura C.C., Mendes P.G., Rocha L.L., Roncalli A.A., Vieira N.F.M., Chianca T.C.M. (2022). Factors associated with stress, anxiety and depression in nursing professionals in the hospital context. Rev. Bras. Enferm..

[bib0008] Batho D. (2022). Experiences of powerlessness and the limits of control in healthcare. Int. J. Philos. Theol..

[bib0009] Belanger C., Chreim S., Bonaccio S. (2024). The experience and implications of meaningless work in the public sector. J. Bus. Eth..

[bib0010] Bell R.L. (2011). Addressing employees’ feelings of inequity: Capitalizing on equity theory in modern management. SuperVision.

[bib0011] Brandes, P., Dharwadkar, R. and Dean, J.W. Jr. (1999) ‘Does organizational cynicism matter? Employee and supervisor perspectives on work outcomes’, paper presented at the 36th Annual Meeting of the Eastern Academy of Management, Philadelphia, pp. 1–33.

[bib0013] Chiaburu D.S., Peng A.C., Oh I., Banks G.C., Lomeli L.C. (2013). Antecedents and consequences of employee organizational cynicism: a meta-analysis. J. Vocat. Behav..

[bib0014] Çivilidağ A., Durmaz Ş., Uslu B. (2025). Investigation of the relationship between organizational cynicism and counterproductive work behaviors: a systematic review and meta-analysis. Front. Psychol..

[bib0015] Demerouti E. (2025). Job demands-resources and conservation of resources theories: how do they help to explain employee well-being and future job design?. J. Bus. Res..

[bib0016] Durrah O., Alalyani W.R., Allil K., Al Shehab A., Al Rawas S., Hubais A., Hannawi S. (2023). The price of silence, isolation, and cynicism: the impact on occupational frustration. Heliyon.

[bib0017] Ejupi V., Squires A. (2024). Exploring influential factors shaping nursing as a profession and science in healthcare system—A systematic literature review. Healthcare.

[bib0018] Elsawy E.H.M. (2023).

[bib0019] Frangieh J., Hughes V., Edwards-Capello A., Humphrey K.G., Lammey C., Lucas L. (2024). Fostering belonging and social connectedness in nursing: evidence-based strategies: a discussion paper for nurse students, faculty, leaders, and clinical nurses. Nurs. Outlook.

[bib0020] Ghaleb B.D.S. (2024). The concept of alienation and alienation in organizations. Pancasila Int. J. Appl. Social Sci..

[bib0021] Healy M., Wilkowska I., Kostera M., Pirson M. (2017). Dignity and the Organization.

[bib0022] Ike O.O., Chuke N.N., Nnamchi O.C. (2024). Organizational cynicism and turnover intention among nurses: do perceived organizational support moderates the relationship. SAGe Open. Nurs..

[bib0023] Kähkönen T., Blomqvist K., Gillespie N., Vanhala M. (2021). Employee trust repair: a systematic review of 20 years of empirical research and future research directions. J. Bus. Res..

[bib0025] Kwon K., Kim T. (2020). An integrative literature review of employee engagement and innovative behavior: revisiting the job demands-resources model. Human Resour. Manage. Rev..

[bib0026] Laschinger H.K.S., Finegan J.E., Shamian J., Wilk P. (2004). A longitudinal analysis of the impact of workplace empowerment on work satisfaction. J. Organ. Behav..

[bib0027] Lee D.Y., Jo Y. (2023). The job demands-resource model and performance: the mediating role of employee engagement. Front. Psychol..

[bib0031] Mohamed R.A., Alhujaily M., Ahmed F.A., Nouh W.G., Almowafy A.A. (2024). Nurses’ experiences and perspectives regarding evidence-based practice implementation in healthcare context: a qualitative study. Nurs. Open..

[bib0032] Mohammed A.N., Hassanein O.A. (2017). The role of alienation within the work environment as an intermediate variable in the relationship between the characteristics of the fundamental self-assessment and job satisfaction: an applied study. Scient. J. Faculty of Commerce, Assiut Univers..

[bib0033] Nakua H., Marsh R. (2025). Integration of developmental and socio-cultural factors that confer risk for mental illness. Neuropsychopharmacology.

[bib0034] Schaufeli W.B. (2017). Applying the job demands-resources model: a “how to” guide to measuring and tackling work engagement and burnout. Organ. Dyn..

[bib0035] Şekerli E.B. (2025). The effect of organizational climate on organizational alienation and destructive deviance behaviors. Yüzüncü Yıl Üniversitesi Sosyal Bilimler Enstitüsü Dergisi.

[bib0037] Uchechukwu A.J. (2024). Strategies for enhancing employee morale in the workplace: a comprehensive approach. Research Invention J. Res. Educ..

[bib0038] Vafeas M., Little E., Vafeas A. (2025). Mitigating work alienation: what can we learn from employee ownership?. Int. J. Hum. Resour. Manage..

[bib0039] Vohra N., Nair N., Sheel R. (2019).

[bib0040] Yao C., McWha-Hermann I. (2025). Contextualizing career development: cultural affordances as the missing link in social cognitive career theory. J. Vocat. Behav..

[bib0041] Yıldız S., Şaylıkay M. (2014). The effect of organisational cynicism on alienation. Procedia - Social and Behav. Sci..

[bib0042] Yu D.S.F., Chen F., Li A., Wong C.W.Y., Chen J., Zhan S., Chan S.W.C., Holroyd E., Chair S.Y., Sim C.Y., Wong W.C.W. (2025). The global landscape of the evolving nurses’ roles and core competency in primary healthcare: an expanding umbrella review. Int. J. Nurs. Stud..

